# Mitochondrial DNA common deletion is not associated with thyroid, breast and colorectal tumors in Turkish patients

**DOI:** 10.1590/S1415-47572009005000102

**Published:** 2010-03-01

**Authors:** Cenk Aral, Mustafa Akkiprik, Handan Kaya, Çigdem Ataizi-Çelikel, Sinan Çaglayan, Gökhan Özisik, Hüseyin Baloglu, Ayse Özer

**Affiliations:** 1 Department of Molecular Biology, Faculty of Science and Arts, Namik Kemal University, TekirdagTurkey; 2 Department of Medical Biology, School of Medicine, Marmara University, IstanbulTurkey; 3 Department of Pathology, School of Medicine, Marmara University, IstanbulTurkey; 4 Department of Endocrinology and Metabolism, Haydarpasa Teaching Hospital, Gulhane Military School of Medicine, IstanbulTurkey; 5 Department of Pathology, Haydarpasa Teaching Hospital, Gulhane Military School of Medicine, IstanbulTurkey

**Keywords:** mitochondrial DNA, common deletion, cancer

## Abstract

Recently, efforts have been focused on mitochondrial DNA changes and their relation to human cancers. Among them, a 4977 bp deletion of mitochondrial DNA, named “common deletion”, has been investigated in several types of tumors, with inconsistent results. In this study, we investigated the presence of the common deletion in tissues from 25 breast, 25 colorectal and 50 thyroid tumors and in the adjacent healthy tissues from Turkish patients. Samples from healthy volunteers were also evaluated for comparison. Two PCR-based methods were used for the detection of the common deletion. First, two pairs of primers were used to amplify wild-type and deleted mtDNA. Then, a highly sensitive nested-PCR was performed, to determine low amounts of deleted genomes. By the first method, wild-type mtDNAs were observed in all samples, but a deletion was observed in only six thyroid samples, by using the nested-PCR method. In conclusion, the mitochondrial common deletion was very rare in our study group and did not appear to be not related with cancer.

Human mitochondrial DNA (mtDNA) is a 16,569-bp circular double-stranded DNA molecule that encodes 13 subunits of the respiratory chain complexes, two ribosomal RNAs, and 22 transfer RNAs. Each nucleated human cell contains a few thousand copies of mtDNA. The somatic mutation rate of mtDNA is presumed to be 10 to 20 times higher than that of nuclear DNA, due to close proximity to the site of reactive oxygen species formation, lack of histones and relatively decreased capacity for repair (Wallace, 1992). There is increasing evidence that mitochondrial gene mutations including large deletions, missense, frameshift mutations, and small deletions/insertions are associated with various types of cancer (Polyak *et al.*, 1998; Fliss *et al.*, 2000; Penta *et al.*, 2001; Modica-Napolitano and Singh, 2004; Aral and Özer, 2007). Among these mutations, a specific deletion of 4977 bp, called the `common deletion' (ΔmtDNA^4977^ mutation), was examined in several human tumors as well as in several metabolic and neurodegenerative disorders, but the results were inconsistent (Maximo *et al.*, 2002; Abnet *et al.*, 2004; Dani *et al.*, 2004; Zhu *et al.*, 2004; Ye *et al.*, 2008). The deletion removes a 4977 bp section of mitochondrial DNA that encompasses five tRNA genes and seven polypeptide genes which encode sub-units of cytochrome c oxidase, complex I and ATPase, between nucleotide positions 8470 and 13447, involving two 13 bp repeats (Soong and Arnheim, 1996). In this study, we investigated the association of the mtDNA common deletion in Turkish patients with breast, colorectal and thyroid tumors.

Paraffin-embedded tissue specimens from primary tumors and matched non-neoplastic adjacent tissues were selected from a total of 100 cancer patients. Briefly, 6 μm thick sections were cut from blocks that had been selected for maximal tumor content. Total DNA was isolated from these thin sections as described by Soong and Iacopetta (1997), following deparaffinization with xylene. Control DNA from blood samples of 49 healthy volunteers was also extracted, using proteinase K digestion followed by phenol/chloroform extraction (John *et al.*, 1991). The study was approved by the ethics committees at Marmara University, School of Medicine and Gulhane Military School of Medicine, Haydarpasa Teaching Hospital.

The mitochondrial common deletion was determined by two different techniques. Primer locations for each technique are schematized in Figure 1a and b. The first method involves two sets of primers: Mitout-F (5'-CCCAACT AAATACTACCGTATGG-3') and Mitout-R (5'-GGCTC AGGCGTTTGTGTATGAT-3') (outside the deletion region); and Mitin-F (5'-CTGAGCCTTTTACCACTCCAG-3') and Mitin-R (5'-GGTGATTGATACTCCTGATGCG-3') (within the deletion region) (Soong and Arnheim, 1996; Maximo *et al.*, 2002) (Figure 1a). In the wild-type mtDNA, only the Mitin primer set yields a PCR product with 142 bp. In cases with the mtDNA common deletion, Mitin primers amplify a 142-bp target sequence, and Mitout primers an aberrant PCR product with 214 bp. PCR amplifications were performed in a total volume of 25 μL containing 200 μmol/L of each dNTP, 12.5 pmol of each of the forward and reverse primers, 50 mmol/L KCl, 10 mmol/L Tris-HCl, (pH 9.0), 1.5 mmol/L MgCl_2_, and 1 U of *Taq* DNA polymerase (Fermentas, Lithuania). Cycling conditions included a single predenaturation step at 94 °C for 5 min, followed by 35 cycles of denaturation at 94 °C for 20 s, annealing at 60 °C for 20 s, elongation at 72 °C for 20 s, and a final incubation at 72 °C for 2 min. The PCR products were visualized on 2% agarose gels stained with ethidium bromide. Secondly, in order to exclude experimental false results and to detect extremely low levels of deletion, we applied a nested-PCR method (Soong and Arnheim, 1996). Primers MT1 (5'-AATTCCCCTAAAA ATCTTTGAAAT-3') and MT3 (5'-AGGCGCTATCAC CACTCTTGTTCG-3') were used for the primary PCR reaction (see Fig. 1b for primer positions). These primers are located just outside the 13 bp repeats where the deletion occurs. One microliter of this primary PCR reaction was then used as a template for the secondary reactions, using primers MT2 (5'-GCGATGAGAGTAATAGATAGGGCTCA GGCG-3') and MT3. Cycling conditions for both primary and secondary reactions were similar to those described for the first method. A 303 bp product was examined by electrophoresis on 2% agarose gels in the samples with the common deletion.

In this study, we searched for the presence of the mitochondrial 4977 bp common deletion in cancerous and healthy tissue samples from 25 breast, 25 colorectal and 50 thyroid cancer patients. Type or locations of the tumors, along with age and gender data of the patients are summarized in Table 1. The mean age of the breast cancer patients, who were all female, was 59 ± 13.8 years. Regarding colorectal cancer patients, the mean age was 62.4 ± 10.2 years, and 12 out of the 25 cases were male. The mean age of the thyroid cancer patients was 37.6 ± 15.8 years, with 27 male and 23 female patients. In all the breast and colorectal cancer samples, adjacent healthy tissues were available, but in seven of the thyroid cancer samples, there was no adjacent healthy tissue. Blood samples were obtained from 49 healthy volunteers (10 male and 39 female) with a mean age of 59.2 ± 12 years.

At first, we successfully amplified mitochondrial genome from all samples using the Mitin primer pair (Figure 1a), which allows amplification of wild-type mtDNA. Then, in order to determine the common deletion, samples including tumors, adjacent healthy tissues and healthy controls were amplified using the Mitout primer pairs, but no common deletion was detected. As previously mentioned by Soong and Arnheim (1996), to detect and measure low levels of deletion in tissues, high sensitivity as well as discrimination between the few molecules of deleted mtDNA and the overwhelming excess of wt-mtDNA molecules are required. These authors suggested concentrating the DNA sample, using radioactively labeled primers or nested-PCR to detect extremely low levels of deletion. In principle, by using the nested-PCR protocol, it is possible to concentrate deleted molecules at the first PCR step and detect single molecules. Thus, all samples (tumor and adjacent healthy tissues and control blood) were then re-amplified using the more sensitive nested-PCR method (see Figure 1b). The mtDNA common deletion was present in only six samples from thyroid cancer patients, whereas this deletion was not observed in the samples from breast or colorectal cancer patients or from healthy volunteers. The deletion-carrying cases are summarized in Table 2. As seen in this table, one case had the mutation in both the cancerous and its adjacent healthy tissues (case ID 69). Two cases had the common deletion in the cancerous tissues, but not in the healthy adjacent tissues (cases 33 and 68). One sample presenting the deletion (case ID 62) in the cancerous tissue had no matching adjacent healthy tissue available to be evaluated.

Previous studies concerning the mtDNA common deletion and cancer have shown inconsistent results. An association of the mtDNA common deletion with a variety of human cancers, including Hürthle cell carcinoma of the thyroid, esophageal squamous cell carcinoma, thyroid and hepatocellular cancers has been reported (Maximo *et al.*, 2002; Abnet *et al.*, 2004). In contrast, several studies showed no significant association with either lung or breast, head and neck, colorectal and gastric carcinomas (Dani *et al.*, 2004; Zhu *et al.*, 2004; Ye *et al.*, 2008). This inconsistency may be due to the laboratory techniques applied and intrinsic features of samples available for each study (*e.g.,* fresh frozen tissue *versus* formalin fixed, age and ethnic background of patients, and so on), as noted by Ye *et al.* (2008). The role of the mtDNA common deletion in carcinogenesis is so far not clear. The mutation may represent a functional disadvantage for the growth of tumor cells (Ye *et al.*, 2008). In the present study, we applied two PCR-based methods to investigate the association of the mtDNA common deletion with breast, colorectal and thyroid tumors in Turkish patients. The mitochondrial DNA common deletion was very rare in either the cancerous or non-tumoral tissues of the patient groups and in healthy controls, suggesting that the deletion is not related with the examined cancer types. The mitochondrial common deletion was detected in six thyroid tumor cases only by the nested-PCR method. This result shows that the method used to detect the mtDNA common deletion is crucial. Availability of both tumoral and non-tumoral adjacent tissues from almost all patients and the use of two different techniques were the strengths of this study. On the other hand, the relatively small number of patients was its main limitation, and further studies with larger cohorts are needed.

**Figure 1:**
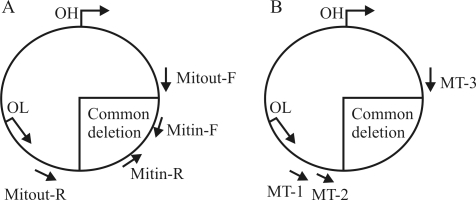
Location of primers in the mitochondrial genome.

## Figures and Tables

**Table 1:** Clinical characteristics of the study population.

	Type of tumor	Mean age ± SD	No. of patients and gender
Breast cancer	Invasive ductal carcinoma	56.4 ± 14.9	13 Females
	Infiltrative ductal carcinoma	56.1 ± 10.1	7 Females
	Infiltrative apocrine carcinoma	60	1 Female
	Inflammatory lobular carcinoma	77	1 Female
	Ductal carcinoma in situ	71	1 Female
	Atypical medullary carcinoma	77	1 Female
	Unknown	77	1 Female

Colorectal cancer	Location of the tumor	Mean age ± SD	No. of patients and gender

	Ascending colon	68 ± 25.4	2 Females
	Descending colon	58 ± 14.8	4 Males
	Cecum	59 ± 9.2	2 Males, 1 Female
	Sigmoid colon	63 ± 8.4	1 Male, 3 Females
	Rectum	58.5 ± 7.8	3 Males, 3 Females
	Transverse colon	69.5 ± 7.7	2 Females
	Recto-sigmoid region	61	1 Female
	Splenic flexure	73.5 ± 0.7	1 Male, 1 Female
	Descending + sigmoid colon	65	1 Male

Thyroid cancer	Type of tumor	Mean age ± SD	No. of patients and gender

	Papillary carcinoma	40.2 ± 17.2	12 Males, 7 Females
	Follicular adenoma	35.9 ± 15.5	11 Males, 8 Females
	Follicular carcinoma	36 ± 17.3*	3 Males, 2 Females
	Medullary carcinoma	38**	1 Male, 1 Female
	Anaplastic carcinoma	50	1 Female
	Oncocytic adenoma	21	1 Male
	Papillary carcinoma + Hashimoto's thyroiditis	26***	2 Females
	Unknown	unknown	1 Female

*: Ages of one male and one female patients with follicular carcinoma were unknown. **: Age of one male patient with medullary carcinoma was unknown. ***: Age of one female patient with papillary carcinoma + Hashimoto's thyroiditis was unknown.

**Table 2:** Summary of the thyroid cancer cases carrying the mtDNA common deletion.

Case ID	Type of tumor	Patient's age and gender	Tumor	Adjacent healthy tissue
33	Follicular adenoma	55, Female	Yes	No
43	Papillary carcinoma	61, Female	No	Yes
62	Papillary carcinoma	53, Male	Yes	Not available
63	Papillary carcinoma	44, Female	No	Yes
68	Papillary carcinoma	29, Male	Yes	No
69	Follicular carcinoma	27, Male	Yes	Yes
